# Non-Oxygenated Sesquiterpenes in the Essential Oil of *Copaifera langsdorffii* Desf. Increase during the Day in the Dry Season

**DOI:** 10.1371/journal.pone.0149332

**Published:** 2016-02-17

**Authors:** Luiz Fernando Rolim de Almeida, Roberto de Oliveira Portella, Jennifer Bufalo, Márcia Ortiz Mayo Marques, Roselaine Facanali, Fernando Frei

**Affiliations:** 1 Department of Botany, Institute of Biosciences of Botucatu, Univ. Estadual Paulista, (UNESP), 18618–970, P.O. Box: 510, Botucatu, Sao Paulo, Brazil; 2 Agronomic Institute, IAC, 13001–970, P.O. Box: 28, Campinas, Sao Paulo, Brazil; 3 Department of Biological Sciences, Univ. Estadual Paulista (UNESP), 19800–000, Assis, Sao Paulo, Brazil; National Taiwan University, TAIWAN

## Abstract

The present study aimed to evaluate the effect of seasonal and diurnal events on the chemical profile of the essential oil obtained from the leaves of *Copaifera langsdorffii* Desf. This study was performed in a Brazilian savanna named Cerrado. We identified the best harvesting period for obtaining the highest amount of compounds used for commercial and industrial purposes. The chemical profile of the essential oils was evaluated by GC-FID and GC-MS, and the results were assessed through multivariate analyses. The data showed that the time of day and seasonal variations affect the quality of the essential oil obtained. Leaves harvested at the end of the day (5:00 pm) in the dry season resulted in richer essential oils with higher amounts of non-oxygenated sesquiterpenes. To the best of our knowledge, environmental conditions induce metabolic responses in the leaves of *C*. *langsdorffii*, which changes the patterns of sesquiterpene production. Therefore, these factors need to be considered to obtain better concentrations of bioactive compounds for pharmacological studies.

## Introduction

Understanding the seasonal events that change the quality of plant volatile compounds is essential for pharmacological studies. Generally, essential oil constituents are influenced by genetic factors and environmental conditions [1]. The terpenoid composition of aromatic plants can be affected by the photoperiod, light intensity, temperature and harvesting season or month [2–5].

The Cerrado biome, a typical savanna-like vegetation in the central region of Brazil, displays seasonal characteristics that promote changes in the water availability and quality and quantity of light [6], which, in turn, result in modifications in the essential oil production and chemical composition of plant products [7]. The Cerrado consists of complex types of vegetation that range from grasslands to woodlands [8]. In addition, this biome is subjected to seasonal events such as the occurrence of dry winters (April to September), which result in changes on the phytochemical profile of essential oils [9] and trigger oscillations on their biosynthesis, yield and composition [10]. Previous studies found a significant impact of the seasonal variation on the essential oil composition of several species such as *Zizyphus spina-christi* (L.) Willd [11], *Ocimum basilicum* L. [12], *Laurus azorica* Franco (Seub.) [13] and *Baccharis dentata* (Vell.) G.M. Barroso [14]. Furthermore, changes in the terpenoid concentration of the essential oils due to diurnal variation have also been observed. A reciprocal regulation between α-cadinol and δ-cadinene occurs in *Calendula arvensis* L. [15], and a lower conversion of mentone to menthol under high temperatures occurred in the essential oils of *Mentha piperita* L. [16]. *Rosa damascena* Mill. flowers modified the essential oil composition during the day, showing an increase of oxides concentrations at 12:00 pm and a decrease during other times of the day [17].

*Copaifera langsdorffii* Desf. (Fabacecae-Ceasalpinoidae), popularly known as copaíba, is a native species of tropical regions of Latin America [18] and is widely distributed in the Brazilian Cerrado and other forests [19]. Specialized metabolites are found in *C*. *langsdorffii* cavities and resiniferous channels, which produce essential oils. The main compounds of the volatile section of the oleoresin are germacrene D, spathulenol and caryophyllene oxide, whereas *ent*-kaurenoids are the major components of the non-volatile section [20]. The oleoresin of *Copaifera* exhibits anti-inflammatory, antimicrobial, larvicidal and antineoplastic pharmacological activities [21]. Moreover, the essential oils of *C*. *langsdorffii* have anti-tumor and healing properties and present gastroprotective activities [18]. Furthermore, the oleoresin of copaíba trees exerts strong antioxidant and anti-inflammatory effects and is composed of several sesquiterpenes and unsaturated diterpene acids [22]. *C*. *langsdorffii* has significant importance in popular traditional medicine because it is widely used in more than 14 categories of body treatments and 55 different health disorders [23]. Regarding the influence of the seasonal and diurnal changes on the essential oil composition, this study provides the first investigation of the qualitative variations in the essential oil composition of *C*. *langsdorffii*. The obtained information will be important for the identification of the optimal chemical profile. Here, we analyzed the seasonal and diurnal variations in the essential oil composition in samples of *C*. *langsdorffii* leaves. Our most significant finding is that there are changes in the levels of major sesquiterpenes.

## Materials and Methods

### Plant materials and experimental design

Leaf samples of three individuals of *C*. *langsdorffii* were collected in 2012 from a semideciduous seasonal forest (SSF; Cerrado physiognomy) in Rubião Júnior, Botucatu, São Paulo State, Brazil (22°53’37.86”S 48°29’22.78”W). A voucher was deposited in the Irina Delanova Gemtchujnicov Herbarium (BOTU) under number 29,274 at the Institute of Biosciences of Botucatu, UNESP. We collected one sample per individual at 6:00 am (early morning) and 5:00 pm (afternoon) at the end of dry season (DS) and wet season (WS). According to the literature [24], the climate of Cerrado in this site shows a dry season (April to September) and a wet season (October to March). Likewise, it presents dry winters and humid summers [25,26]. *C*. *langsdorffii* leaves were harvested packed in paper bags and placed at a drying in a forced-air drier at 35˚C for 72 h.

To study the influence of these seasonal variations, we considered the dry season from July to October with an average temperature of 20.2˚C and an average rainfall of 52.88 mm; we collected the samples on October 03^rd^. The wet season included the months of January through March with an average temperature of 25.0˚C and an average rainfall of 582.88 mm; we collected the samples on March 27^th^. During the sample collecting day, at early morning (6:00 am), in dry season the light intensity was 55 μmol m^-2^s^-1^ and in wet season was 3 μmol m^-2^s^-1^. On the other hand, during afternoon (5:00 pm) the light intensity was 147 μmol m^-2^s^-1^ in wet season and 173 μmol m^-2^s^-1^ in dry season. The water balance, that shows periods of water excess (EXC) and water deficit (DEF) before collecting days can be seen in supporting information (S1 Fig).

### Ethics statement

Necessary permits for the field study was obtained by Dr. Luiz Fernando Rolim de Almeida to Ministério do Meio Ambiente—MMA, Instituto Brasileiro do Meio Ambiente e dos Recursos Naturais Renováveis–IBAMA (the Brazilian Environmental Institute), Sistema de Autorização e Informação em Biodiversidade—SISBIO, process n° 26541–1; authentication code n° 84241528. The location where the plants were collected were not privately owned or protected in any way and the field studies did not involve endangered or protected species.

### Extraction and analysis of essential oils

The essential oils were extracted by hydrodistillation using a Clevenger apparatus. Each sample consisted of 50 g of dried leaves distilled for 120 min. The essential oil yield was calculated by weight as (g) of oil per (g) of dry material and expressed as percentage of oil in the dried leaves. The quantitative analysis of the essential oil compounds was obtained by gas-chromatograph GC (Shimadzu GC-2010) equipped with flame ionization (GC–FID) and using a DB-5 (J and W Scientific; 30 m × 0.25 mm × 0.25 mm × 0.25 μm) capillary column. Helium was used as the carrier gas, and the injector temperature was 240˚C.; the detector temperature was 230˚C, the gas flowed at 1.0 mL/minute, and it was split 1/20. The temperature program was set at 60˚C-200˚C at 3˚C/minute and 200˚C-240˚C at 15˚C/minute. The injection temperature was 230˚C. The injection volume was 1.0μL and consisted of a sample preparation that included 1.0 μL of essential oils added into 1.0 mL of ethyl acetate. The qualitative analysis of the essential oil compounds was performed on a gas-chromatograph (GC) coupled to a mass spectrometer (MS; GC–MS; Shimadzu QP-5000) operating at a MS ionization voltage of 70 eV. The chromatograph was equipped with a fused silica capillary column DB-5 (J and W Scientific; 30 m × 0.25 mm × 0.25 μm), and helium was used as the carrier gas. The temperature program was 60˚C-240˚C at 3˚C/minute. The injection temperature was 220˚C. The injection volume was 1.0 μL of sample preparation that contained 1.0 μL of essential oils added into 1.0 mL of ethyl acetate. The compounds were identified by comparing their mass spectrum to those of the database on the GC-MS (NIST 62.lib and Wiley 139.Lib) literature [27] and retention indices [28]. The retention index (RI) was obtained by injection injecting a mixture of n-alkanes (C9-C24) and applying the equation of the method of [29]. The data were analyzed with three repetitions per period of data collection.

### Statistical analysis

We performed multivariate and box plot analyses following the values of major compounds. In addition, an agglomerative hierarchical cluster (AHC) was performed following a 118 squared Euclidean distance dissimilarity matrix, which was calculated by the Ward’s linkage. We also constructed a matrix of correlation by a principal component analysis (PCA) and to identify whether a significant difference exists between the means of two groups—6:00 am and 5:00 pm—during the wet and dry seasons we used Student's t-test (p ≤ 0.05).

## Results and Discussion

### Chemical profile of essential oils

The phytochemical profile of the essential oils from *C*. *langsdorffii* leaves showed 17 compounds, namely 13 non-oxygenated sesquiterpenes and four oxygenated sesquiterpenes (Table 1). The essential oil yield was an average from 0.03–0.05% without significance difference between seasonal and diurnal variation (Table 1). The major compounds were germacrene D, β-caryophyllene, bicyclogermacrene, spathulenol, caryophyllene oxide and α-cadinol (Table 1). A comparable result was previously reported by [20], who found β-caryophyllene, germacrene D, spathulenol and caryophyllene oxide in the essential oils from *C*. *langsdorffii* leaves. Similar results were reported by [30] for the leaves, fruits, trunk and roots of *C*. *langsdorffii*, all of which contained β-caryophyllene and caryophyllene oxide in their volatile fraction. Germacrene D has been previously reported to be one of the main components of *Copaifera officinalis* (Jacq.) L. among the 25 sesquiterpenes found in the leaf tissues of this plant [31]. Furthermore, other authors have reported the chemical composition of *Copaifera* species [32–34] and revealed the same compounds detected in the *C*. *langsdorffii* essential oils in this study.

**Table 1:** Average of essential oil composition and yield of *Copaifera langsdorffii* Desf. from a semideciduous seasonal forest at 6:00 am and 5:00 pm during the wet and dry seasons.

Compounds (%)	Wet season			Dry season			RI exp	RI lit	Method of identification
	6:00 am	5:00 pm	t-test	6:00 am	5:00 pm	t-test			
*Non-oxygenated sesquiterpenes*									
α-Copaene	0.24 ± 0.04	0.34 ± 0.13	ns	tr	1.49 ± 0.62	-	1373	1376	MS, RI
β-Elemene	0.79 ± 0.29	0.82 ± 0.14	ns	tr	1.21 ± 0.50	-	1389	1391	MS, RI
β –Caryophyllene	5.47 ± 6.19	2.15 ± 0.79	ns	1.07 ± 0.77	9.02 ± 0.17	*	1416	1418	MS, RI
γ-Gurjunene	tr	tr	-	tr	0.29 ± 0.05	-	1426	1432	MS, RI
γ-Elemene	tr	tr	-	tr	tr	-	1436	1439	MS, RI
α –Humulene	0.88 ± 0.89	0.40 ± 0.07	ns	tr	1.30 ± 0.14	-	1450	1454	MS, RI
γ-Muurolene	0.56 ±0.26	0.54 ± 0.10	ns	0.40 ± 0.37	1.31 ± 0.09	*	1476	1477	MS, RI
Germacrene D	14.11 ± 14.88	7.73 ± 4.98	ns	4.01 ± 4.44	17.99 ± 4.35	*	1478	1480	MS, RI
Bicyclogermacrene	4.72 ± 5.33	2.33 ± 1.14	ns	1.51 ± 1.86	5.68 ± 0.91	*	1495	1494	MS, RI
α –Muurolene	0.54 ± 0.55	0.26 ± 0.06	ns	0.47 ± 0.48	1.38 ± 0.09	*	1497	1499	MS, RI
γ-Cadinene	0.63 ± 0.27	0.51 ± 0.09	ns	0.79 ± 0.54	1.31 ± 0.17	ns	1512	1513	MS, RI
δ-Cadinene	3.07 ± 2.12	1.80 ± 0.67	ns	1.75 ± 1.75	2.72 ± 0.33	ns	1522	1524	MS, RI
Germacrene B	1.48 ± 0.64	1.72 ± 0.09	ns	1.36 ± 0.31	1.80 ± 1.49	ns	1557	1556	MS, RI
*Oxygenated sesquiterpenes*									
Spathulenol	27.39 ± 16.31	35.65 ± 3.54	ns	25.90 ± 12.28	12.55 ± 11.11	ns	1576	1576	MS, RI
Caryophyllene oxide	14.49 ± 7.03	16.32 ± 3.63	ns	16.55 ± 5.56	7.40 ± 1.14	ns	1581	1581	MS, RI
Humulene epoxy II	2.12 ± 1.46	2.59 ± 0.04	ns	2.63 ± 0.40	1.73 ± 0.08	*	1604	1606	MS, RI
α-Cadinol	3.25 ± 0.99	3.2 ± 0.46	ns	7.93 ± 4.54	5.24 ± 0.94	ns	1650	1653	MS, RI
Non-oxygenated sesquiterpenes	32.49	18.60		11.36	45.50				
Oxygenated sesquiterpenes	47.25	57.76		53.01	26.92				
Ratio (Non-oxygenated sesquiterpenes/Oxygenated sesquiterpenes)	0.69	0.32		0.21	1.69				
Yield of essential oils (%)	0.04 ± 0.02	0.04 ± 0.03	ns	0.03 ± 0.01	0.05 ± 0.04	ns			

RIexp.: experimental Retention Index. RI lit.: Retention Index found on literature. tr: trace amount (≤ 0.001). MS, identification based on comparison of mass spectra. RI, identification based on retention index.

*p ≤ 0.05 (statistical significance).

ns: non-significant.

The weather conditions, particularly light intensity and precipitations, affected the essential oil composition. During the dry season, the average precipitation was 52.88 mm, and this value reached 582.88 mm during the wet season. A decrease in the light intensity was observed during the wet season, with an average of 286 μmol m^-2^s^-1^, whereas the dry season presented an average of 665.67 μmol m^-2^s^-1^. As a result, the main compounds of non-oxygenated sesquiterpenes and oxygenated sesquiterpenes showed higher or lower amounts depending on the average light intensity and precipitation during early mornings and afternoons.

Previous studies reported that weather parameters, such as atmospheric temperature and rainfall, influence the oil content and composition of several aromatic plants [35]. In agreement with those results, our study demonstrates that the essential oil composition of *C*. *langsdorffii* was affected by environmental factors, such as rainfall and light intensity. In addition, a decrease in the average temperature observed during the dry season could also alter the pattern of sesquiterpene production.

In Table 1, the ratio of non-oxygenated sesquiterpenes versus oxygenated sesquiterpenes showed an inversion during the dry season at the end the day (1.69). A ratio of non-oxygenated sesquiterpenes/oxygenated sesquiterpenes higher than 1.0 reports an elevated level of non-oxygenated sesquiterpenes observed at 5:00 pm during the dry season. It have been suggested by others studies [17,15,16] that variations in the terpene levels throughout the day constitute evidence of the up and down regulations of a specialized metabolism influenced by environmental factors.

The non-oxygenated sesquiterpenes, i.e., germacrene D, β-caryophyllene and bicyclogermacrene, showed a higher trend during the early harvesting of leaves and decreased thereafter in the wet season. Therefore, during the dry season, those compounds presented a higher trend at 5:00 pm (Table 1). The oxygenated sesquiterpenes, i.e., spathulenol and caryophyllene oxide compounds, showed a higher trend at 5:00 pm during the wet season and at the early morning harvest during the dry season. α-Cadinol showed a higher tendency at 6:00 am and opposite trend after that time during the dry season (Table 1). In the present study, the non-oxygenated sesquiterpenes decreased in the late harvest during wet season; however, a reverse trend was observed during dry season.

The most important environmental abiotic factors that influence plant growth and development are water availability and temperature [24], as well as light also can contribute on modifying plant metabolism [36–38]. According with literature, 20% of sesquiterpene emissions are impacted by temperature and 80% by temperature and light together [39,40]. *Lippia origanoides* Kunth showed higher oxygenated sesquiterpenes levels during wet season and a predominance of oxygenated monoterpenes and monoterpenes hydrocarbons during dry season [41].

Based upon observation, our results show that multiple environmental factors change the specialized metabolism on plants, resulting in production of different terpenes. This suggests that chemical profile showed on this study is result of accumulating environmental signals on leaves such as water availability variation, the raising of light hours during the day and temperature. Furthermore, it should be noted that signaling of the environment in plant metabolism can be seen in our results, mostly by the inversion of the ratio of non-oxygenated sesquiterpene and oxygenated sesquiterpenes at the end of dry days.

The quality of the essential oils of *C*. *langsdorffii* was affected by the time of the day and seasonal variations. For instance, the two major compounds, i.e., germacrene D and spathulenol, showed opposite results according to seasonal and diurnal variations. Germacrene D (non-oxygenated sesquiterpene) showed an increasing tendency during the early mornings of the wet season as well as during the dry season at 5:00 pm (Table 1). In contrast, spathulenol (oxygenated sesquiterpene) presented an increasing trend during the dry season at 6:00 am and during the wet season at 5:00 pm (Table 1). This result suggests that the light intensity and water availability can influence sesquiterpene biosynthesis and production. Moreover, this variation in the chemical profile of essential oils can alter their medicinal activity because essential oils offer a complex mixture of substances and, generally, the major compounds are the ones responsible for their biological properties [10].

### Multivariate analysis of major compounds

**Fig 1:**
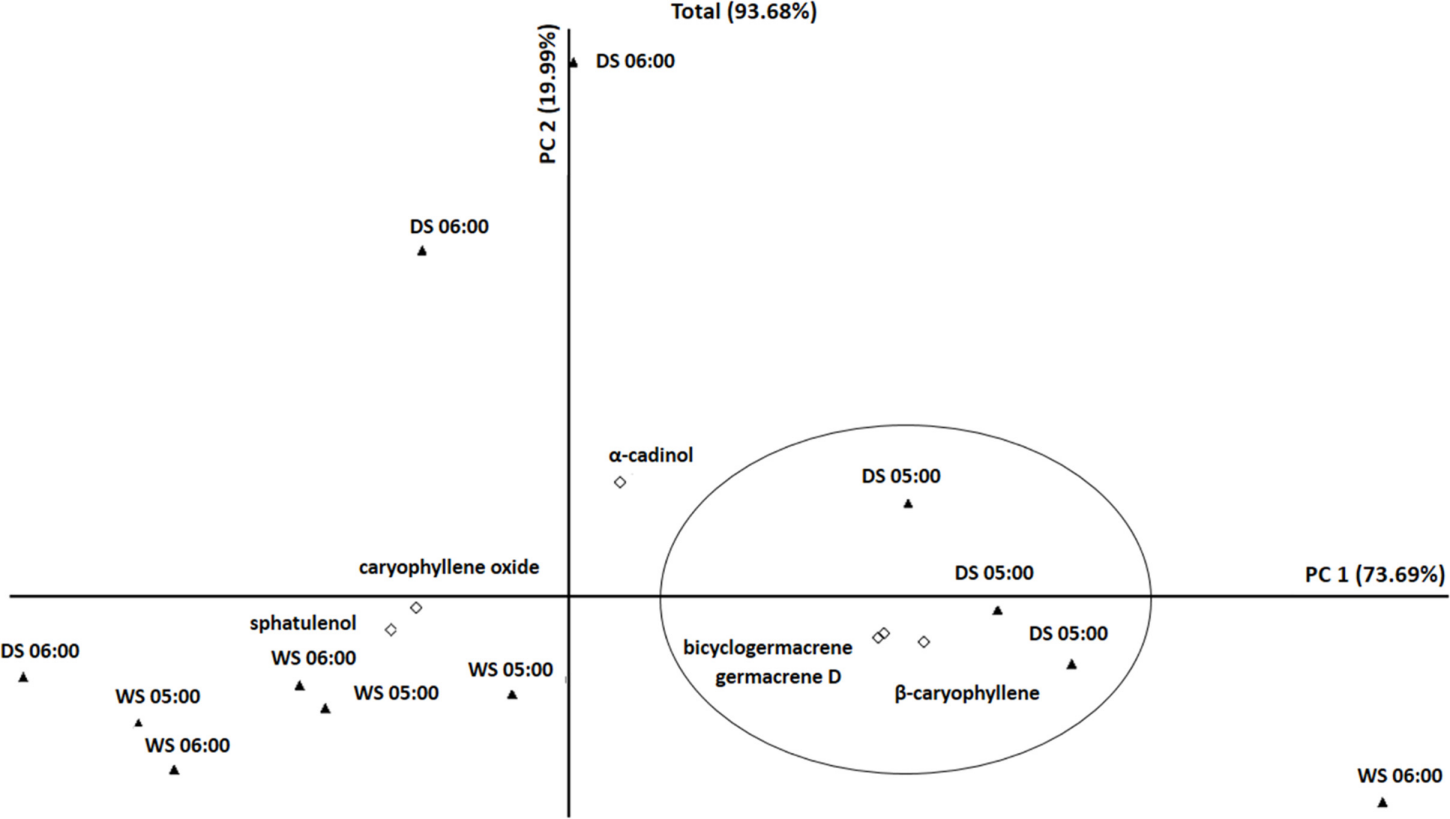
PCA of the main compounds present in the essential oil of *Copaifera langsdorffii* Desf from a semideciduous seasonal forest at 6:00 am and 5:00 pm during the dry season (DS) and the wet season (WS).

We applied a principal component analysis (PCA) to assess the composition of the essential oils from *C*. *langsdorffii* and to identify a possible correlation between the main compounds and the seasonal and diurnal variations (Fig 1). The principal factorial plane accounts for 93.68% of all major compounds. In addition, the PC1 axis (73.69%) shows that the non-oxygenated sesquiterpenes (germacrene D, bicyclogermacrene and β-caryophyllene) were positively correlated with the afternoon (5:00 pm) harvest of the dry season. The oxygenated sesquiterpenes (spathulenol and caryophyllene oxide) were correlated with both harvests (6:00 am and 5:00 pm) of the wet season. In contrast, α-Cadinol was the only compound that was not correlated with seasons and harvesting periods.

**Fig 2:**
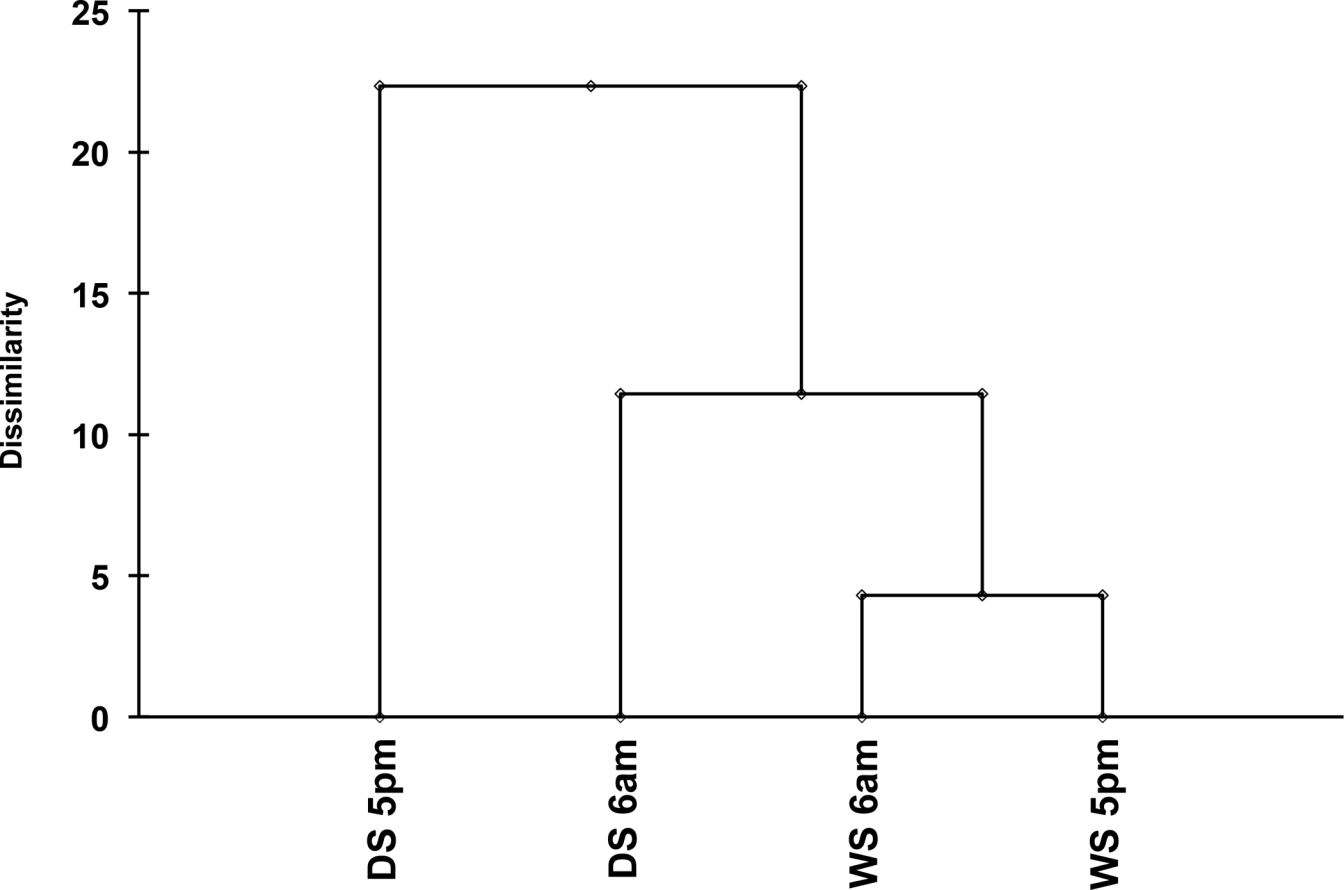
Agglomerative Hierarchical Clustering (AHC) calculated with the Ward’s linkage and Euclidean distance, which correlates the essential oil data from a semideciduous seasonal forest. Wet Season at 6:00 am (WS 6am) and Wet Season at 5:00 pm (WS 5pm)—Group 1; Dry Season at 6:00 am (DS 6am)—Group 2; Dry Season at 5:00 pm (DS 5pm)—Group 3.

**Fig 3:**
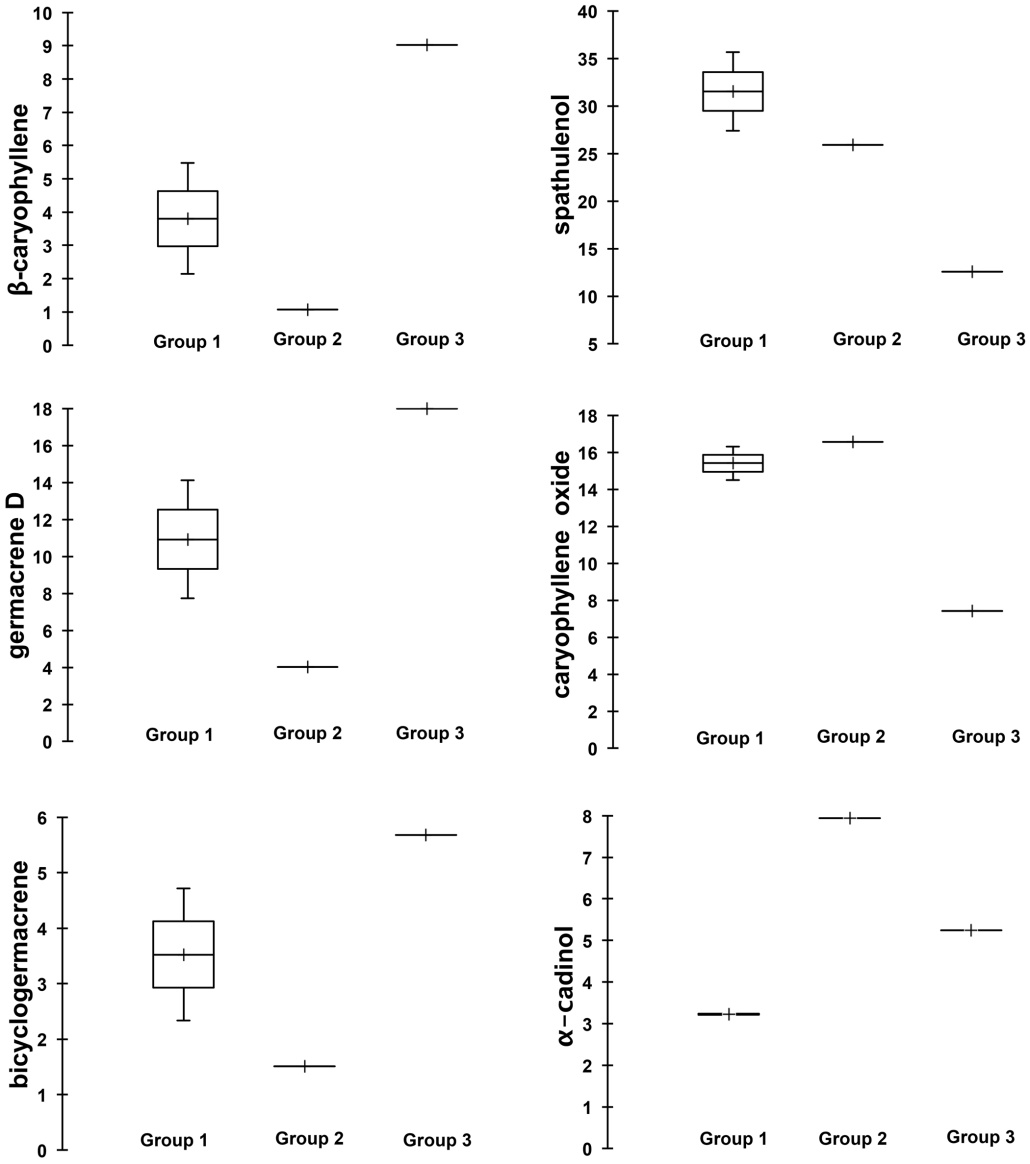
Box plot analyses of the major compounds (%) present in the essential oil from leaves of *Copaifera langsdorffii* Desf. β-Caryophyllene, germacrene D and bicyclogermacrene (non-oxygenated sesquiterpenes); spathulenol, caryophyllene oxide and α-cadinol (oxygenated sesquiterpenes). Wet season at 6:00 am and wet season at 5:00 pm—Group 1; dry season at 6:00 am—Group 2; dry season at 5:00 pm—Group 3.

The pattern of the major essential oil compounds of *C*. *langsdorffii* was categorized into three groups through cluster and box plot analyses (Figs 2 and 3, respectively). The first group consisted of the compounds present during the wet season at 6:00 am and the wet season at 5:00 pm; the second group consisted of the compounds present during the dry season at 6:00 am; and the third group consisted of the compounds present during the dry season at 5:00 pm (Fig 2). Our data show that β-caryophyllene, germacrene D and bicyclogermacrene had the highest values in group 3. Likewise, spathulenol and caryophyllene oxide had the highest values in group 1 and 2 (Fig 3). Furthermore, the PCA analysis reveals a correlation between the oxygenated sesquiterpenes and the wet season as well as between the 5:00 pm harvests of the dry season with the non-oxygenated sesquiterpenes.

The light wavelength and intensity well as the exposure time are essential features for the biosynthesis of metabolites in plant cells [42]. The flux of terpenes found in *Citrus sinensis* (L.) Osbeck, *C*. *clementii*, and *Picea abies* (L.) H. Karst. followed diurnal variations controlled by temperature, with maximum concentrations during midday and minimal concentrations during the evenings and mornings [43]. The peak emissions of isoprene occurred during mid-afternoon hours. Instead, the α-pinene-to-β-pinene ratio were high during early morning and late afternoon [44]. Moreover, α-cadinol and caryophyllene oxide presented the highest concentrations at 7:00 pm in *Pycnocycla spinosa* Decne. ex Boiss. [42], and high temperatures influenced the release of *trans*-caryophyllene [45]. Sesquiterpenes had the highest levels at 6:00 pm in August, and the main compound of the essential oils from leaves of *Cistus monspeliensis* L. was caryophyllene oxide [46]. The highest levels of carvacrol in the essential oils of *Origanum onites* L. occurred at 10:00 am and the lowest at 06:00 am [47]. The authors reported that the thymol content was higher at 12:00 am (23.76%) and lower at 08:00 pm. These authors also showed a biosynthetic correlation between thymol and carvacrol based on the dynamic levels of these compounds. In the present study, germacrene D, β-caryophyllene and bicyclogermacrene were upregulated during the dry season and downregulated during the wet season. In addition, spathulenol and caryophyllene oxide were upregulated during the wet season and downregulated during the dry season. Therefore, we now have a better understanding of the metabolic adjustments during injuries are regulated by the environmental status. Furthermore, it is necessary to understand the association between the sesquiterpene metabolism and the effects of these compounds on the plants.

Additionally, we believe that the leaf protection against herbivory and pathogens occurs as a physiological response against biotic stresses. For instance, *C*. *langsdorffii* maintains part of its leaves during stressful events, and it is classified as a perennial species [19]. Hussain et al. found in *Ocimum basilicum* L. a seasonal trend in the oxygenated and non-oxygenated sesquiterpene production [12]. These authors observed high amounts of oxygenated sesquiterpenes during winter and a minimal production of sesquiterpene hydrocarbons and, an opposite trend, during the summer. According to our results, we believe that stressful events, such as water scarcity, increase the production of non-oxygenated sesquiterpenes. Therefore, these compounds display important medicinal properties, as reported by [48], who showed the antimicrobial activity of germacrene D present in the essential oil of *Ageratum fastigiatum* (Gardner) R.M. King & H. Rob. Similarly, the essential oils from *Murraya koenigii* (L.) Spreng. showed antibacterial effects due to the presence of β-caryophyllene [49]; in addition, the essential oils of *Callicarpa japonica* Thunb. were found to exert phytotoxic effects due to the presence of bicyclogermacrene and germacrene D [50]. Furthermore, oxygenated sesquiterpenes also displays biological properties, for instance, spathulenol found in essential oils of *Nepeta glomerata* Montbret et Aucher ex Bentham that showed cytotoxic and antibacterial activities [51] and it was the major constituent (4.5%) in *Achillea cretica* essential oil responsible for antimicrobial activity [52]. Therefore, research on the properties of the compounds found in the oleoresin of *Copaifera* species [21] and studies regarding the environmental influence on the terpenoid metabolism are essential for obtaining the highest concentrations of natural products with beneficial properties to human health.

## Conclusions

This study demonstrates that the diurnal variation in light intensity and water accumulation during the different seasons control the sesquiterpene levels of the essential oil composition of *C*. *langsdorffii*. Harvests performed at 5:00 pm in dry days resulted in oils of *C*. *langsdorffii* with a higher percentages of non-oxygenated sesquiterpenes. These data support the importance of monitoring the metabolic responses of plants caused by environmental factors. This type of ecological research revealed a method for obtaining the maximum concentrations of bioactive compounds used for medicine.

## Supporting Information

S1 Fig Water balance per month at semideciduous seasonal forest, Cerrado physiognomy. The wet season represent an accumulation of water, as well as dry season a period of deficiency of water availability. EXC–water excess; DEF–water deficit.
(DOC)
