# Supplementary material for: Non-Oxygenated Sesquiterpenes in the Essential Oil of Copaifera langsdorffii Desf. Increase during the Day in the Dry Season
Source: PLoS One. 2016 Feb 17;11(2):e0149332. doi: 10.1371/journal.pone.0149332 (PMC4757570; doi:10.1371/journal.pone.0149332)
Supplement: S1 Fig — The wet season represent an accumulation of water, as well as dry season a period of deficiency of water availability. EXC–water excess; DEF–water deficit. (DOC) [file pone.0149332.s001.doc]

**Supporting information**


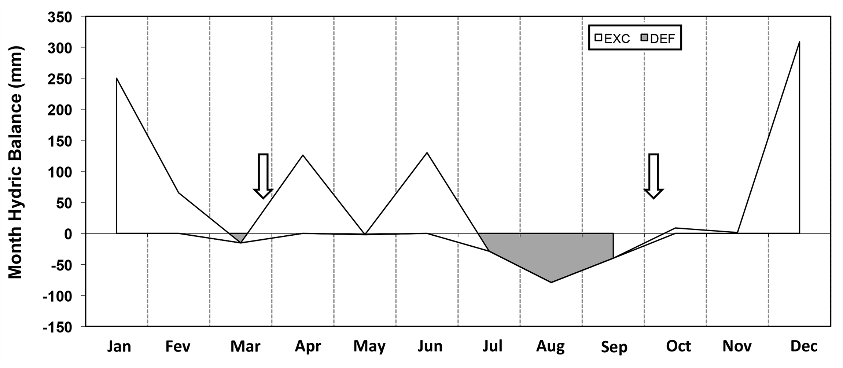


**S1 Fig. Water balance per month at semideciduous seasonal forest, Cerrado physiognomy. The wet season represent an accumulation of water as well as dry season a period of deficiency of water availability. EXC – water excess; DEF – water deficit.**
